# Emerging Roles for 3′ UTRs in Neurons

**DOI:** 10.3390/ijms21103413

**Published:** 2020-05-12

**Authors:** Bongmin Bae, Pedro Miura

**Affiliations:** Department of Biology, University of Nevada, Reno, NV 89557, USA; bae.bbm@nevada.unr.edu

**Keywords:** 3′ UTR, alternative polyadenylation, local translation, RNA-binding protein, RNA-sequencing, post-transcriptional regulation

## Abstract

The 3′ untranslated regions (3′ UTRs) of mRNAs serve as hubs for post-transcriptional control as the targets of microRNAs (miRNAs) and RNA-binding proteins (RBPs). Sequences in 3′ UTRs confer alterations in mRNA stability, direct mRNA localization to subcellular regions, and impart translational control. Thousands of mRNAs are localized to subcellular compartments in neurons—including axons, dendrites, and synapses—where they are thought to undergo local translation. Despite an established role for 3′ UTR sequences in imparting mRNA localization in neurons, the specific RNA sequences and structural features at play remain poorly understood. The nervous system selectively expresses longer 3′ UTR isoforms via alternative polyadenylation (APA). The regulation of APA in neurons and the neuronal functions of longer 3′ UTR mRNA isoforms are starting to be uncovered. Surprising roles for 3′ UTRs are emerging beyond the regulation of protein synthesis and include roles as RBP delivery scaffolds and regulators of alternative splicing. Evidence is also emerging that 3′ UTRs can be cleaved, leading to stable, isolated 3′ UTR fragments which are of unknown function. Mutations in 3′ UTRs are implicated in several neurological disorders—more studies are needed to uncover how these mutations impact gene regulation and what is their relationship to disease severity.

## 1. Introduction

3′ untranslated regions (UTRs) enable post-transcriptional control to provide spatiotemporal regulation of gene expression within a cell. Recognition of *cis*-elements by RNA-binding proteins (RBPs) drives tight modulation of gene expression by altering mRNA degradation rates, driving subcellular localization of mRNA, and regulating translation efficiency. These changes ultimately impact the spatiotemporal dynamics of protein synthesis. In this review, the roles that 3′ UTRs play in controlling mRNA dynamics in the nervous system are discussed, along with new emerging roles for 3′ UTRs that go beyond their roles in mRNA localization and translation.

## 2. *Cis*-Acting Roles for 3′ UTRs in Neuronal Gene Regulation

### 2.1. miRNA Regulation

**Figure 1:**
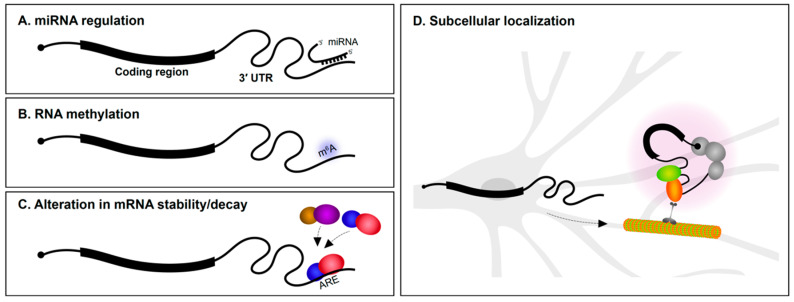
Post-transcriptional regulation via 3′ untranslated regions (3′ UTRs). 3′ UTRs mediate post-transcriptional gene regulation via (**A**) miRNA interactions, (**B**) RNA methylation, (**C**) regulating mRNA stability/decay by interaction with RNA-binding proteins (RBPs; illustrated as colored balls), and (**D**) imparting subcellular localization within neurons to regions such as dendrites and axons, where they can undergo local translation. ARE, AU-rich element.

The 3′ UTR is particularly well understood as the target region for microRNA (miRNA) regulation (Figure 1A). Several miRNAs are specifically expressed in the nervous system and have roles in neural development and maintenance. During the early stages of neural specification and differentiation, gene regulatory networks that establish neuronal identity are regulated by miRNAs [1,2,3]. miRNAs also regulate axon outgrowth/pathfinding and dendritogenesis [3,4,5]. This is achieved in part by miRNA suppression of mRNAs encoding a wide variety of cytoskeletal and signaling proteins [6,7,8,9]. In fully mature neurons, miRNAs are involved in synaptic plasticity [10,11,12] and the regulation of circadian rhythms [13]. Given these important roles for miRNAs in neurons, it is not surprising that alterations in miRNA expression is implicated in neurodegenerative disorders, such as Alzheimer’s disease (AD) and Parkinson’s disease [14,15,16], and neuropsychiatric problems, including depression, anxiety, schizophrenia, and autism spectrum disorder [17,18,19]. Along the same lines, mutations in 3′ UTRs encoding miRNA seed sites have also been associated with several disorders [20,21,22].

### 2.2. RNA Methylation

Methylation of DNA is well understood to regulate chromatin dynamics and transcription. In recent years, it has emerged that methylation of RNA also impacts gene regulation (Figure 1B). While there are many types of RNA modifications, N6-methyladenosine (m^6^A) is one of the most abundant and the best characterized to date [23]. Transcriptome-wide analysis revealed that m^6^A modifications are particularly enriched in 3′ UTRs [24,25]. m^6^A modifications play roles in axon guidance, neurogenesis, neural survival, and synaptic function [26,27]. m^6^A modifications appear to be particularly important for control of mRNA translation. YTHDF1 (YTH N6-methyladenosine RNA binding protein 1) is a m^6^A reader protein. By binding to m^6^A marked regions, predominantly in 3′ UTRs, YTHDF1 regulates protein synthesis in response to neuronal activity. Mice lacking YTHDF1 display impaired hippocampal synaptic transmission and defects in learning and memory, demonstrating the key role for RNA methylation in the nervous system [28]. 

### 2.3. mRNA Stability and Translational Control 

The rate of mRNA decay is influenced by several determinants, including poly(A) tail length and 3′ UTR sequence content. RBP interactions with 3′ UTRs are important determinants of mRNA stability (Figure 1C). AU-rich elements (AREs) located in 3′ UTRs were first reported to mediate mRNA decay of cytokine transcripts [29]. AU-rich binding proteins bind AREs and can stabilize or promote degradation of the mRNA. For instance, the KH-type splicing regulatory protein (KSRP or KHSRP) and HuD (ELAV-like protein 4) are RBPs that bind to an ARE in the 3′ UTR of the GAP-43 (*Gap43*) mRNA. Although both proteins target the same region, they employ antagonistic regulation of GAP-43 mRNA—KSRP enhances its turnover during axonal outgrowth of hippocampal neurons [30], whereas HuD stabilizes it in select neuronal populations [31]. mRNA stability influences not only the number of times an mRNA can be translated, but also impacts mRNA localization since the transcript must resist degradation in order to be subcellularly localized.

## 3. 3′ UTR-Mediated Subcellular Localization of mRNAs within Neurons

Ever since the first findings of asymmetric localization of mRNAs in ascidian eggs [32], subcellular localization of transcripts in polarized cells has been of great interest. In neurons, mRNAs are found in axons, dendrites, and synapses [33,34,35,36] (Figure 1D). The number of subcellularly localized transcripts identified in neurons continues to grow as a result of advances in transcriptomics and methods to isolate dendrites and axons from soma. Most mRNA localization transcriptome studies have been performed in cultured primary explants/neurons [37,38,39,40] and immortalized neuronal-like cells such as Neuro2A and CAD cells [41] (see Box 1). Methods to physically isolate long axons/processes from cultured tissues/cells include the use of compartmentalized chambers and membrane inserts. These allow axons to be separated from soma for downstream RNA analysis. We are now aware that hundreds to thousands of mRNAs are found in axons or dendrites of both the peripheral and central nervous systems [39,42,43], and that the transcriptome of each neuronal subcompartment is unique. It is more challenging to identify dendrite/axon localized mRNAs using sequencing approaches in vivo. Laser microdissection of dendrite-enriched regions followed by RNA-Seq has been successfully performed in various systems including rat brain slices [42]. It is clear from such studies that many mRNAs are found in dendrites and axons.

Box 1 **APA trends in cultured neuronal-like cells versus neurons.**

Selection of the proper biological system is a key for studying the dynamics of alternative 3′ UTR usage in neurons. Most previously reported studies have employed either (1) immortalized neuron-like cell lines, (2) isolated neurons, (3) stem cell/induced pluripotent stem cell derived neurons, or (4) tissues. A key benefit of using cell lines are their malleability. For instance, the properties of 3′ UTRs and localizing potentials of Tau and GAP-43 have been largely studied in P19 and PC12 cells [44,45,46]. Much of the research focusing on miRNA binding to 3′ UTRs has been performed in Neuro2A cells [47,48] and the localization of alternative last exon 3′ UTR isoforms in neurites was studied using differentiated CAD and Neuro2A cells [41]. Expression of alternative 3′ UTR isoforms can vary widely between mouse tissues and cell lines. Several examples are shown in this figure. RNA-Seq reads were aligned using HISAT2 [49], processed using SAMtools [50], and tracks visualized at the last two exons of the *Calm1*, *Rac1*, and *Ranbp1* genes using Integrated Genomics Viewer [51]. Note the changes in read coverage pertaining to the alternative long 3′ UTRs. Gene models in light blue represent un-annotated transcript isoforms. SRA accession numbers are noted.

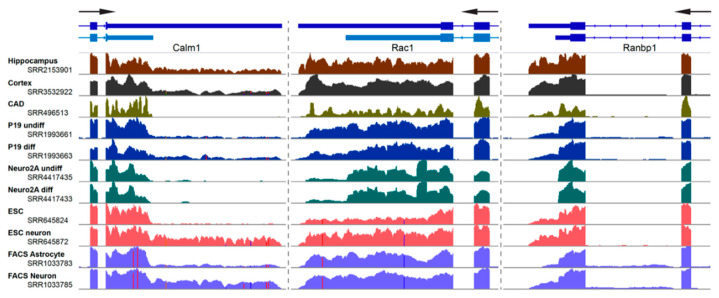

### 3.1. RNA Localizing Cis-Elements

The study of mRNA subcellular localization determinants has focused on *cis*-acting sequence elements in mRNAs and the RBPs that bind them. The subcellular localization of mRNAs in neurons has often been attributed to 3′ UTR sequences [52,53]. Since the identification of a localizing *cis*-element in the β-actin 3′ UTR, known as zipcode, and its *trans*-acting RBP partner, zipcode-binding protein (ZBP; as known as IMP1) [54,55,56], considerable attention has been focused on determining 3′ UTR *cis*-elements and RBP *trans*-factors influencing mRNA localization [39,43,57]. Although some motif enrichment has been observed, efforts to find a universal “zipcode” sequence governing mRNA localization has been unsuccessful. Sequences identified as localization elements are largely unique to each gene. Thus, mRNA localization elements cannot currently be identified by a motif-searching bioinformatics approach (for examples of localizing *cis*- and *trans*- elements, refer to [53]).

Assessing the role of 3′ UTRs in mRNA localization has heavily relied on the use of 3′ UTR reporter assays. For this type of experiment, the 3′ UTR sequence of interest is subcloned downstream of a reporter, such as a fluorescent protein (FP), or adjacent to mRNA reporter motif, such as MS2 bacteriophage coat protein-binding sequences, to observe the effect of the 3′ UTR on localization (For more experimental approaches, see Box 2). Then, a minimal localizing element can be identified by testing the effect that 3′ UTR deletions or mutations have on reporter mRNA axonal or dendritic localization. Using reporter systems, 3′ UTR sequence determinants for localization have been identified for CaMKIIα (*Camk2a*), β-actin (*Actb*), GAP-43, Importinβ1 (*Kpnb1*), and *Rgs4* mRNAs [58,59,60,61,62]. 

Only a few studies have uncovered the impact of 3′ UTR sequences on localization using loss of function approaches in vivo. The localizing role of the 3′ UTR of CaMKIIα in mice was confirmed by inserting a heterologous poly(A) site into the endogenous locus to prevent full length 3′ UTR generation. This approach successfully prevented mRNAs from being localized in dendrites [63]. 3′ UTR-mediated localization of β-actin mRNAs in vivo was identified using a heterologous reporter construct harboring different 3′ UTR sequences [52]. The heterologous reporter transgenic approach showed that the 3′ UTR of β-actin directed expression of the reporter gene to axons [52]. Localization of *Bdnf* alternative 3′ UTR mRNAs have been investigated in vivo through similar approaches as well (see Section 4.2. Neural functions of long 3′ UTR mRNA isoforms). Recent technical advances in genome editing have facilitated 3′ UTR deletions with increased precision and speed in animal models. For example, the CRISPR-Cas9 (Clustered regularly interspaced short palindromic repeats-Cas9) system was used to delete part of the mTOR (*Mtor*) 3′ UTR which was found to impair local translation in dorsal root ganglion (DRG) neurons [64]. 

All these results suggest that 3′ UTRs impact the localization of transcripts in neurons in vitro and in vivo. There is plenty of evidence for certain sequences to be sufficient for mRNA localization in neurons, but there is still scant evidence that they are necessary. For example, although the 3′ UTR of β-actin was found to be sufficient to drive expression of the reporter gene in axons [52], there are still no published studies, to our knowledge, showing the impact of deleting zipcode sequences from the genome on endogenous β-actin mRNA localization. Precise genome-editing techniques such as CRISPR/Cas are now commonplace, so in the coming years we expect new studies that characterize transgenic animals with deletions in single putative localization elements. 

mRNA localizing elements can be more complicated than simply a primary nucleic acid sequence. For instance, the spatial arrangement of localization elements within the 3′ UTR can impact localization [65]. Localizing elements can also be comprised of structural features, such as G-quadruplex motifs [66]. Although identification of RNA structural motifs is challenging, it is becoming more feasible with the advent of new RNA structure probing methods that allow for identification of RNA secondary structures in cells [67]. Perhaps the continued search for single localization elements is misguided? It is emerging that multiple elements with redundant functions within 3′ UTRs might impact localization—3′ end sequencing analysis of axons and cell bodies found that multiple elements spanning across 3′ UTRs influence subcellular localization as opposed to single regions [42]. It should also not be forgotten that localization elements can also be found in 5′ UTRs [68,69,70]. Perhaps many localized mRNAs rely on the presence of multiple, redundant localization elements in both 5′ UTRs and 3′ UTRs.

Box 2 **Experimental approaches to study localization of mRNAs.**

Fluorophore-labeled probe-based methods, such as fluorescence in situ hybridization (FISH), have improved in situ detection of mRNAs in fixed neurons in term of resolution and sensitivity compared to previous in situ hybridization methods, allowing single molecule RNA detection. Largely, two types of RNA FISH methods are available. The first method is based on usage of multiple oligo probes each harboring a fluorophore that target a same single RNA molecule (e.g., Stellaris®) [71]. The other type of method is based on amplification of fluorescence signal by in situ biochemical reactions, such as rolling-circle based method (e.g., OligoMix®) [72], branched DNA method (e.g., RNAscope®) [73], and primer-exchange reaction based method (e.g., SABER-FISH) [74]. Advanced techniques, such as MCP-FP (MS2 bacteriophage coat protein-FP), λN-FP (N protein of bacteriophage λ-FP), RCas9-FP (dead RNA Cas9-FP), and fluorescent RNA aptamer system, have allowed visualization of RNA trafficking in live cells. Co-expression of a MCP-FP or λN-FP protein construct and a reporter construct containing phage protein binding motif sequence upstream of 3′ UTR of interest allowed tracking of mRNA localization in live cells [75,76]. Simultaneous delivery of RCas9-FP and target-specific single guide RNA allowed binding of the Cas9 to the mRNA of interest and visual tracking of endogenous mRNAs in live cells [77]. Use of fluorescent RNA aptamers, such as Peppers, has overcome dimensional limitations of FP tethering techniques and enhances signal-to-noise ratios allowing improved in vivo tracking [78].

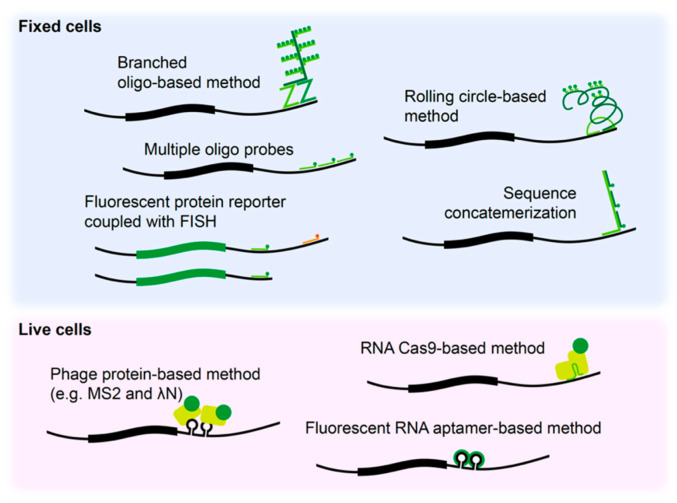

### 3.2. Role of RBPs in mRNA Localization 

How do 3′ UTR sequences in mRNAs drive localization to axons and dendrites? An attractive hypothesis is that the mRNAs are actively transported. The trafficking of RBP–mRNA complexes, referred as messenger ribonucleoproteins (mRNPs), has been associated with cytoskeleton-based transport systems [79,80,81]. Motor proteins usually lack RNA-binding domains, thus, it is widely considered that the localizing transcripts associate with RBPs and accessory proteins to indirectly interact with motor proteins [80]. ZBP1 (which interacts with the β-actin 3′ UTR) has been reported to interact with the motor protein KIF5A (kinesin heavy chain isoform 5a) and Myosin Va [82,83]. ZBP1-containing mRNPs mediate dynamic movement and dendritic localization of β-actin mRNA [56]. 

Alternatively, an mRNA might be found in a particular subcellular compartment because of passive diffusion and long half-life conferred by 3′ UTR–RBP interactions [84]. A transcriptome-wide assessment of mRNA stability in somata and neuropil compartments of rat Cornu Ammonis (CA1) region suggested that neuropil localized 3′ UTR isoforms have longer half-life compared to non-localized 3′ UTR mRNA isoforms [42]. This might be, at least partially, due to increased GC-content and predicted secondary structure elements in neuropil localized RNAs which confer structural stability [42]. The same study also has revealed that neuropil localized 3′ UTR mRNA isoforms are enriched for neuronal RBP motifs that have been associated with mRNA localization [42]. RBP-mediated selective transport and mRNA stability-dependent localization mechanisms need not be mutually exclusive since many RBPs are multi-functional and could mediate both active localization and stability.

### 3.3. RNA Granules 

There are emerging roles for RNA granules in neuronal mRNA localization and translation control [85]. RNA granules are membraneless intracellular structures that possess liquid-like properties to support their dynamic organization and serve as a means of compartmentalizing and accelerating biochemical reactions [86,87,88]. These granule structures are self-assembled through liquid–liquid phase separation. One of the components that facilitates liquid–liquid phase transition and formation of granules are proteins that contain low complexity domains. These domains induce liquid–liquid phase transition through self-aggregation (recently reviewed in [89,90]). Thus, it has been proposed that RBPs with low-complexity domains might control both selective recruitment of RNAs and the formation of RNA granules through phase transition [91,92]. For instance, FMRP (fragile X mental retardation protein) and FUS (fused in sarcoma) proteins induce phase transition and translational control in mRNPs [93,94]. An interesting characteristic of mRNA components of these granules is that these mRNAs contain significantly longer 3′ UTR sequences and are enriched in binding motifs for the granule RBPs [91], suggesting 3′ UTR contents impact RNA granule dynamics.

A recent study showed that protein constituent of RNA granules also mediates the interaction between RNA granules and moving membranous organelles to drive microtubule-dependent long-distance transport of RNAs. Annexin A11 is a protein containing a low-complexity domain and membrane binding domain. Through these domains, Annexin A11 mediates association of RNA granules with lysosomes to control RNA localization in neurons [95]. In vivo functional roles in neurons for phase transition have recently been uncovered. TIAR-2 (TIA-1/TIAL RNA binding protein homolog), an RNA-binding-domain-containing TIA family protein found in *Caenorhabditis elegans*, was found to mediate phase transitioning and formation of liquid-like granules. Mutating prion-like domains in TIAR-2 mitigates granule formation and impaired axon regeneration [96]. How RNA granules regulate gene expression and impact nervous system functions will certainly be an area of intense investigation for years to come. 

## 4. Alternative Polyadenylation Generates Long 3′ UTRs in Neurons

**Figure 2:**
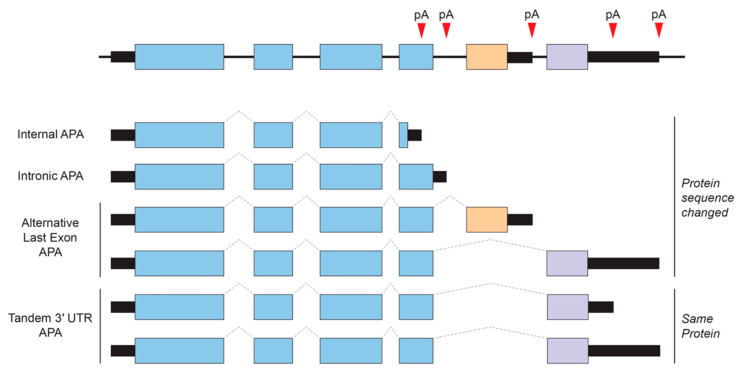
Types of alternative polyadenylation (APA). APA generates alternative 3′ ends for a given gene. Tandem 3′ UTR APA is the most common and does not cause changes in protein-coding regions, whereas other APA events that are sometimes linked to regulated alternative splicing can result in alternative last exons that have different coding and 3′ UTR content. pA, poly(A) site.

Alternative polyadenylation (APA) is an RNA processing mechanism that allows the generation of alternative mRNAs with distinct 3′ ends from a single gene through the selective usage of alternative polyadenylation (poly(A)) sites. In mammals, at least 50–70% of protein-coding genes undergo APA, and many of these alternative 3′ UTRs show tissue-specificity of expression [97,98,99]. APA can be largely classified into two categories (Figure 2). One produces alternative protein isoforms through internal/intronic polyadenylation or through alternative last exon splicing choices. The other type of APA occurs within the 3′ UTR via tandem poly(A) sites and generates transcript isoforms that harbor the same coding sequences (CDS) and thus only differing in their 3′ UTR content. 

Early microarray studies and work on individual genes revealed a bias among brain tissues for expressing higher levels of long versus short 3′ UTR mRNA isoforms for genes expressed in multiple tissues [100,101,102]. Later studies using RNA-Seq revealed the transcriptome-wide catalog of these neural-specific 3′ UTR lengthening events (see Box 3 for bioinformatics methods to quantify alternative 3′ UTR usage). In *Drosophila*, nearly 400 genes were found to express previously unannotated long 3′ UTR isoforms in head samples and late stage embryos [103]. Some of these transcripts had 3′ UTRs of staggering length, dwarfing the length of the protein-coding region of the mRNA. For example, the long 3′ UTR of *mei-P26* was found to express an 18.5 kb long 3′ UTR [103]. Investigation of long 3′ UTRs in neural tissues of mouse and human yielded similar findings of neural-specific enhancement of long 3′ UTRs with thousands of previously unannotated long 3′ UTR isoforms being identified [104]. 

A bias for longer 3′ UTRs in the nervous system appears to be attributed to expression in neurons as opposed to other neuronal cell types such as astrocytes, microglia, and oligodendrocytes [105,106,107]. This raises two questions: (1) What is the mechanism that leads to longer 3′ UTRs in neurons? (2) Do these longer 3′ UTR mRNAs have specific neural functions?

Box 3 **Quantification of alternative 3′ UTR mRNA isoforms using standard RNA-Seq data.**

Although RNA-Seq has become a routine procedure, the identification and quantification of alternative 3′ UTR isoforms using RNA-Seq data presents many challenges. Primarily, two types of detection algorithms are currently used: (1) de novo detection of APA isoforms based on the read density changes and (2) reliance on annotated or reported 3′ ends. De novo detection-based methods do not rely on 3′-end sequencing data or previously reported 3′ ends, thus providing unique advantages. Change-Point is a 3′ UTR APA detection software that identifies APA events between two conditions based on read density changes [108]. It compares the ratio of mapped reads in the common 3′ UTR region and the ratio of the extended 3′ UTR region between two samples and the identification of exact APA site is based on the location where ratio change is the maximum. Dynamic analyses of alternative polyadenylation from RNA-Seq (DaPars) is a de novo 3′ UTR APA detection software that uses similar read density-based approach but quantifies dynamic APA events between two or more experimental conditions and allows identification of novel long 3′ UTRs [109]. In contrast to the two previous methods, APAtrap allows identification of multiple APA sites. First, novel 3′ UTR ends are identified by assessing the coverage of RNA-Seq reads through the annotated 3′ end and further downstream regions. Once the most 3′ end is defined as the distal poly(A) site, then potential proximal poly(A) sites are identified using the read density changes [110]. Given the expansion of 3′ end databases, APA events can also be reliably detected using methods based on annotated Poly(A) sites. Quantification of APA (QAPA) estimates the expression of alternative 3′ UTR isoforms using annotated poly(A) sites. First, it builds a reference library comprising all the reported 3′ UTR sequences. Then, RNA-Seq reads are mapped to the 3′ UTR library using alignment-free algorithm. APA usage is quantified by the ratio of isoform expression to the sum of the expression of all detected 3′ UTR isoforms from its cognate gene. This method identifies multiple APA events and is able to process multiple datasets at a time [105]. Significance analysis of alternative polyadenylation using RNA-Seq (SAAP-RS) is another method based on Poly(A) database. The 3′ UTR regions are split into upstream (UP) and downstream (DN) regions. RNA-Seq read distribution is assessed in the UP and DN region to determine the relative expression difference [107].

### 4.1. APA Mechanisms Underlying Long 3′ UTR Expression in Neurons 

The mRNA 3′ end processing machinery includes cleavage and polyadenylation specificity factors (CPSF), cleavage stimulation factors (CstF), cleavage factors (CF I and II), poly(A)-binding protein (PABP), poly(A) polymerase (PAP), and RNA polymerase II (Pol II), among other proteins [111]. One of the mechanisms that mediates APA selection involves these 3′ end processing factors. APA patterns have been associated with the expression levels of CstF64 [112,113,114] and CFI [115,116]. These studies suggested that the concentration of the specific processing factors might define the usage of poly(A) signals leading to the cell/tissue-specific 3′ UTR expression patterns. The speed or pausing of Pol II can also influence APA, by providing sufficient time to recruit 3′ end processing factors and to process the poly(A) site [117]. A slower RNA Pol II mutant showed preferential selection of proximal poly(A) sites in *Drosophila* [118]; however, this effect was limited to non-neural tissues, suggesting a different regulatory mechanism at play in neurons [119]. 

**Figure 3:**
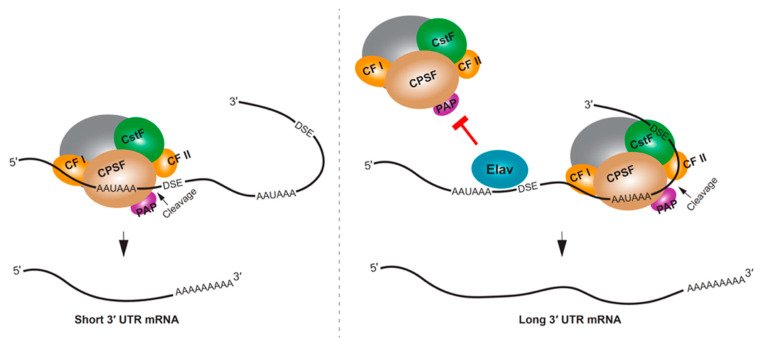
Mechanisms of neural APA in *Drosophila*. The neuronal RBP Elav regulates APA by binding to target mRNA proximal poly(A) site region to block the 3′ end processing machinery. This promotes the usage of downstream distal poly(A) sites. Consequently, neuronal tissues of *Drosophila* selectively express APA isoforms with longer 3′ UTRs. DSE, downstream element; AAUAAA is shown as a representative polyA signal.

RBPs can influence APA by competing with the cleavage and polyadenylation machinery for access to the poly(A) site. One of the most striking examples of RBP regulating 3′ UTR extension is the neural-specific RBP, Elav (embryonic lethal abnormal visual protein) in *Drosophila*. *Drosophila* embryos lacking Elav were found to lack long 3′ UTR isoforms for several genes, and the ectopic expression of Elav in non-neural tissues resulted in mRNAs with longer 3′ UTRs [120]. A putative mechanism for this regulation is through Elav binding to the proximal poly(A) site, thus competing with 3′ end processing, and causing the selection of a downstream poly(A) site [120] (Figure 3). In addition to directly binding RNA, an additional role for Elav binding to DNA promoter sequences has been demonstrated, and this might also be required for Elav regulation of APA in *Drosophila* neurons [121]. In mammalian neural tissues or neuron-like cells, the Elav homolog ELAVL3 (ELAV-like protein 3), along with NOVA2 and FUS have been shown to impact 3′ UTR APA [122,123,124]. 

### 4.2. Neural Functions of Long 3′ UTR mRNA Isoforms

Given the relevance of 3′ UTRs in controlling the subcellular localization of mRNAs, one hypothesis of long 3′ UTR function has been that it localizes due to the extra 3′ UTR sequences not found in the short 3′ UTR counterpart. This phenomenon was first characterized for the long 3′ UTR mRNA isoform of *Bdnf*. Loss of the long 3′ UTR mRNA isoform was found to impair dendritic localization of the mRNA and cause defects in spine morphology and synaptic plasticity [125]. 

Based on the findings for *Bdnf*, groups have speculated that a function of the alternative long 3′ UTRs might be to impart subcellular localization. However, transcriptomic studies have failed to identify a strong bias for long 3′ UTR isoforms to be preferentially localized compared to short 3′ UTR counterparts [42,43]. Although many 3′ UTR isoforms showed subcellular localization bias, a significant number of the alternative 3′ UTR transcripts reside in the same soma or neuropil subcompartment [42]. Another study actually found evidence that shorter 3′ UTR isoforms are more abundant than long 3′ UTR isoforms in neurites of mouse embryonic stem cell (ESC)-derived neurons [126]. These results suggest that long 3′ UTRs may not generally confer axon/dendrite localization.

## 5. Functional Relevance of Local Translation in Neurons 

Regardless of the roles for alternative 3′ UTR isoforms, the notion of localized mRNAs undergoing local translation in both dendrites and axons is now well appreciated [127,128]. Local translation allows precise spatiotemporal regulation of protein expression. The specific role of localized mRNA translation has been explored largely in three contexts: (1) neuron development and neurite outgrowth, (2) synaptic function in mature neurons, and (3) neurite maintenance and regeneration.

Localization and translation of mRNAs in growing neurons has been explored especially for cytoskeleton-related genes to support neurite pathfinding and asymmetrical organization. Tau, an axonal microtubule-associated protein encoded by the *Mapt* gene, is one mRNA that localizes mainly into axons, and its role in establishing neuronal polarity might rely on axonal translation [129,130,131]. *Rhoa* mRNAs axonally localize in embryonic DRG neurons in a 3′ UTR-dependent manner. During cytoskeletal rearrangement of the growth cone, 3′ UTR-dependent local translation of RhoA is necessary to induce growth cone collapse in response to guidance cue Sema3A (Semaphorin 3A) [132]. 

The ability of axonally or dendritically localized mRNAs to undergo local translation is critical for synaptic functions. Synaptic plasticity, including homeostatic scaling, is a process that is dependent on translation of axonal and dendritic proteins [133,134,135,136]. Synaptic concentration of ion channels, such as Kv1.1 (*Kcna1*), is regulated through 3′ UTR-dependent localization and translation in hippocampal neurons [137]. Synaptic rearrangement is another cellular process that takes advantage of local translation. Matrix Metalloproteinase 9 (*Mmp9*), an endopeptidase that regulates the pericellular environment, is localized in a 3′ UTR-dependent manner and undergoes translation when hippocampal neurons are activated [138]. Neurogranin (*Nrgn*) undergoes local translation, during which FMRP exerts regulation through its 3′ UTR, and plays a role in synaptic plasticity and memory encoding [139]. All these examples lead to an open conclusion that synaptic function requires local translation of specific transcripts. 

Local translation is relevant for neuron maintenance and regeneration—it is implicated in retrograde signaling, mitochondrial function, and transcription factor activation related to the axon maintenance and regeneration. Several proteins involved in injury-induced retrograde singling pathways have been shown to be locally translated. Some examples include 3′ UTR mRNA isoforms of *Ranbp1* (Ran-specific binding protein 1) and Importinβ, which are involved in nuclear export and import, respectively [59,140]. Mitochondria-related proteins also undergo local translation to support axon maintenance [141]. Nerve growth factor-induced local translation of myo-inositol monophosphatase-1 (*Impa1*) is critical for nuclear CREB (cyclic AMP-responsive element-binding protein) activation and prevention of axonal degeneration [142]. Local protein synthesis and turnover during the axon regeneration is considered to be regulated by various pathways dependent on mTOR, p38 mitogen-activated protein kinase, and caspase [143]. mTOR regulates local translation of other mRNAs through its own local translation in injured axons and regulates other retrograde injury signaling molecules [64]. 

Multiple techniques have been developed in order to study local translation. Some of these techniques rely on reporter or fusion protein system and some on metabolic or genetic labeling (see Box 4). Studying local translation in vivo in animals, however, is more challenging. Some metabolic labeling techniques, such as FUNCAT (fluorescent noncanonical amino acid tagging), BONCAT (bio-orthogonal non-canonical amino acid tagging), and OP-Puro (O-propargyl-puromycin) labeling, enable global tagging of the newly synthesizing proteins in vivo through specific diet or injection of metabolic analogs [144,145,146]. These techniques, however, have not been applied to in vivo nervous systems and do not allow transcript (3′ UTR isoform)- or gene-specific analysis but rather confirm robustness of translation upon specific physiological contexts.

Box 4 **Studying local translation.**

Investigating the mRNA sequences regulating mRNA localization and local translation has largely relied on the use of fluorescent protein (FP) reporters. One of the caveats of using FP to study localized translation is the ability to freely diffuse within cells. Myristoylation of FP (Myr-FP) anchors the protein with membrane limiting its diffusion thus providing spatial information on the translating protein [147]. Technical advances have allowed visualization of local translation at increased temporal resolution. The photoconvertible FP, Kaede, fusion techniques have been used to study local translation. Kaede emits green fluorescence until it is cleaved by UV-induction then it emits red fluorescence. Newly synthesized proteins can be detected by green fluorescence after UV treatment [137]. One can label newly synthesizing proteins by pulse-chase application of amino acid orthologs and click-chemistry through BONCAT or FUNCAT methods [144,145]. These methods allow both purification of newly synthesized proteins and in situ visualization. Puromycin labeling-based methods incorporate Puromycin, an analog of aa-tRNA, into newly synthesized proteins to inhibit amino acid polymerization. Thus, puromycin conjugates can be used to visualize de novo protein synthesis [148]. Similarly, puromycylation of a specific protein can be detected in situ by Puro-PLA (proximity ligation assay) through in situ coincidental detection of anti-Puromycin and anti-POI antibodies [149]. Some techniques provide detection power both for mRNAs and newly synthesized proteins allowing bonafide detection of localized translation, although they still require multiple tagging strategies. Translating RNA Imaging by Coat protein Knock-off (TRICK) methods takes advantage of phage coat proteins and its binding sequences, i.e., PP7 and MS2, to visualize the displacement of PP7-GFP by translating ribosomes and co-detection of its cognate mRNA by MS2-RFP [150]. Single-molecule Imaging of NAscent PeptideS (SINAPS) uses reporter constructs containing multiple SunTag epitope for visualization of translation and MCP binding sequence for mRNA detection [151]. Similarly, nascent chain tracking (NCT) uses multiple FLAG epitope tags and antibody-based detection of translating proteins and MCP system to visualize mRNAs [152].

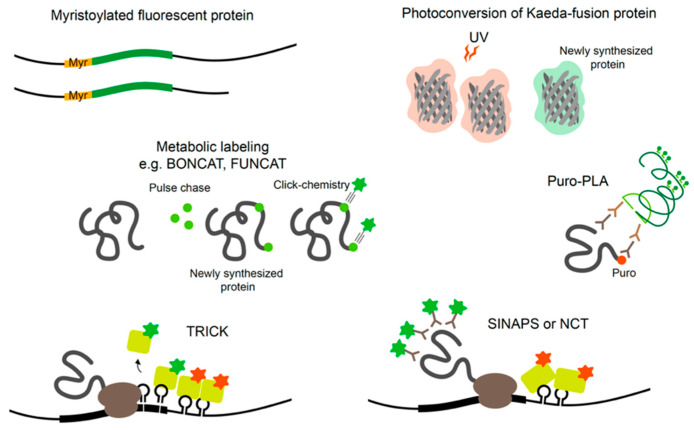

## 6. Distinct Functions for Locally Synthesized Proteins

**Figure 4:**
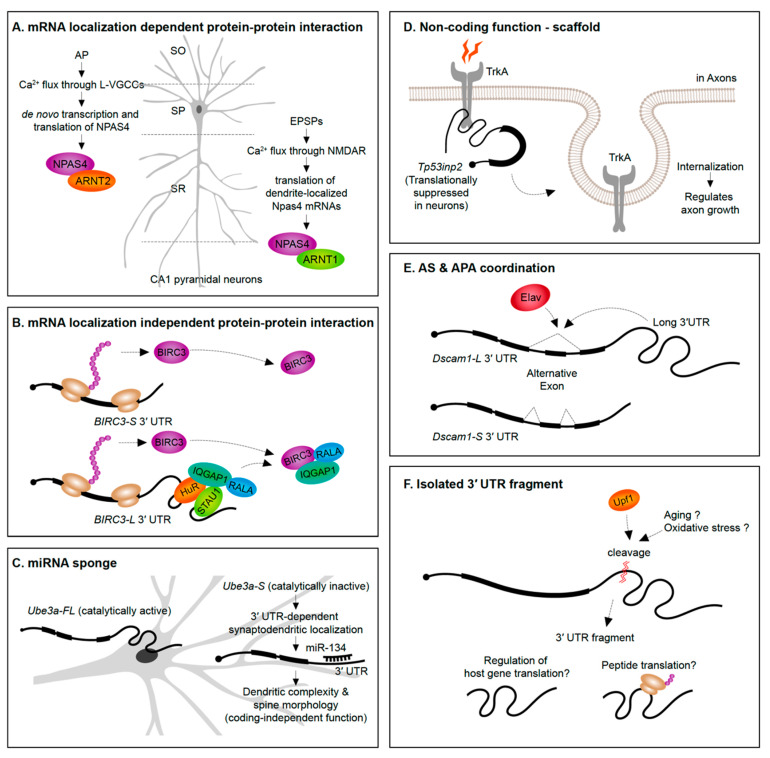
Newly emerging roles for 3′ UTRs. (**A**,**B**) mRNA 3′ UTRs can serve as scaffolds for the interactome of their host proteins. (**C**) 3′ UTRs can serve a non-coding role as a sponge for miRNAs. (**D**) Translationally inactive 3′ UTRs mRNA isoforms can impact internalization of receptor proteins and influence axon growth. (**E**) 3′ UTR sequences can act as mediators of alternative splicing on the same pre-mRNA. (**F**) Isolated 3′ UTR fragments cleaved from mRNAs might regulate host gene translation and/or encode peptides. AP, action potential; L-VGCC, L-type voltage-gated calcium channel; EPSP, excitatory postsynaptic potential; NMDAR, NMDA receptor; SO, stratum oriens; SP, stratum pyramidale; SR, stratum radiatum; CA1, cornus ammonis 1.

A straightforward way to envision the role of mRNA subcellular localization is that it can provide rapid translation to generate a protein required in a spatial region of interest at a precise time. For instance, translation of β-actin mRNAs localized into axonal growth cones increases the protein levels in the same growth cone region to support axon guidance [153]. An emerging theme, however, is that protein synthesis in specific subcellular regions exposes the newly synthesized proteins to distinct proteomic repertories. In the case of the *Npas4* gene, NPAS4 (neuronal PAS domain protein 4) proteins synthesized from the dendrite-localized and soma-localized transcripts interact with distinct protein partners (Figure 4A). Soma-localized *Npas4* transcripts undergo de novo translation upon neuron activation through L-type voltage-gated calcium channels (L-VGCCs) in the Stratum pyramidale layer (soma region) of hippocampus. These soma-originated NPAS4 proteins associate with ARNT2 (aryl hydrocarbon receptor nuclear translocator 2) and act as nuclear transcription factors. This mechanism is distinct from the heterodimerization of NPAS4 triggered by excitatory postsynaptic potential (EPSP) induction. In the latter case, dendritically localized *Npas4* and *Arnt1* mRNAs undergo local translation, form heterodimers, and are translocated together to the nucleus to bind transcription promoter or enhancer regions [68]. This example not only distinguishes differential means of inducing NPAS4 expression upon distinct neuronal signals but also emphasizes the importance of compartmentalization of mRNAs and its translation to modulate the protein–protein interactome. 

Alternative 3′ UTRs can recruit RBPs to increase the likelihood that an RBP interacts with the nascent protein as it is translated, thus impacting protein–protein interactions. For instance, the RBP HuR (ELAV-like protein 1) interacts with the long 3′ UTR of CD47 (leukocyte surface antigen CD47) to act as an intermediate scaffold mediating SET-dependent localization of CD47 proteins to the plasma membrane [154]. Another example is the long 3′ UTR isoform of BIRC3 (baculoviral IAP repeat containing 3) that, unlike the short isoform, recruits a unique set of proteins. The long 3′ UTR of BIRC3 recruits a protein complex composed by IQGAP1 (IQ motif containing GTPase-activating protein 1), RALA (Ras-related protein), STAU1 (Staufen 1), and HuR, to promote their interaction with nascent BIRC3 proteins [155]. Interestingly, the 3′ UTR isoforms do not differently drive mRNA or protein localization, suggesting that the long 3′ UTR-dependent function of BIRC3 is not localization or local translation. Instead, the long 3′ UTR of BIRC3 serves as a scaffold RNA to enhance protein–protein interactions (Figure 4B). These studies were performed in non-neuronal cell lines—in the future it will be interesting to see if analogous examples emerge from studies of alternative 3′ UTR isoforms expressed in neurons.

## 7. Beyond Localization and Translational Control

In addition to the conventional roles for 3′ UTRs in regulating mRNA stability, localization, and translational control, several new functions of 3′ UTRs have emerged in recent years.

### 7.1. Coding-Independent Functions of 3′ UTRs

Evidence is emerging that alternative 3′ UTR isoforms have functions in *trans*, independent from influencing the protein-coding functions of the gene they are transcribed from. *Ube3a1* is an essential gene implicated in Angelman syndrome and Autism spectrum disorders. An activity induced *Ube3a1* transcript is alternatively polyadenylated at intron 11 to encode an isoform lacking the catalytic domain. This alternative 3′ UTR isoform is synaptodendritically localized. Although knockdown of *Ube3a1* impaired dendritic complexity and spine morphology, this was not due to the protein-coding capacity of the transcript. *Ube3a1* RNA containing a frameshift mutation or *Ube3a1* 3′ UTR alone was able to fully rescue the dendritic phenotypes acting as endogenous miRNA sponge [156] (Figure 4C).

*Tp53inp2* is a transcript highly enriched in axons of sympathetic neurons that has a protein coding-independent function. *Tp53inp2* is able to translate into proteins in other tissues, but it is translationally suppressed in neurons due to its long 3′ UTR, suggesting its unique role as a non-coding mRNA in sympathetic neurons. Despite the lack of translation, *Tp53inp2* mRNA is involved in TrkA (tropomyosin-related kinase receptor A) receptor internalization and downstream signaling, and phenotypes can be rescued by non-translatable *Tp53inp2* transcript [157] (Figure 4D). 

The precise molecular mechanisms underlying non-coding roles for alternative 3′ UTR isoforms remain to be illuminated. More examples should continue to emerge given the new feasibility of isoform specific deletions using CRISPR-Cas9 technologies, and isoform specific knockdown approaches such as short hairpin RNAs (shRNAs), antisense oligonucleotides, and CRISPR-Cas13 [158].

### 7.2. Coordination of Alternative Splicing and APA

Potential crosstalk between alternative splicing and APA has been suggested in the past since several RBPs have documented roles in regulating both of these pre-mRNA processing events [120,123,159]. An example of a 3′ UTR isoform being associated with a particular alternative splicing event of the same gene has been recently discovered. In *Drosophila*, the expression of the long 3′ UTR isoform of the *Dscam1* gene was found to be coupled to the skipping of an upstream exon. The neuronal RBP Elav was found to induce both the expression of the long 3′ UTR and skipping of an upstream exon. Long-read sequencing on the Oxford Nanopore MinION platform demonstrated the tight connection between the alternative splicing and APA events—long 3′ UTR mRNAs in adult heads were found to always skip the upstream exon. Moreover, shRNA-mediated knock-down of exon skipping transcripts abolished the long 3′ UTR isoform of *Dscam1* and vice-versa. This connectivity of an exon skipping event and an alternative long 3′ UTR could be explained simply by the co-regulation by Elav. However, it was found that Elav could only exert its influence on the exon skipping event when the *Dscam1* long 3′ UTR was present [160] (Figure 4E). 

What is the mechanism of 3′ UTR-mediated regulation of exon skipping? It is possible that the 3′ UTR acts as a splicing factor delivery system. In such a model, intramolecular interactions between upstream introns and the 3′ UTR might occur, allowing RBPs bound to the 3′ UTR to interact with upstream splice sites. Or perhaps 3′ UTR sequences bind to upstream intronic sequences that are important for splicing, and block association of spliceosome components? Future work is needed to determine if coupling of 3′ UTR choice to upstream alternative splicing events is widespread and whether it occurs in human neurons. To date, widespread coupling of 3′ UTR selection to alternative exons have not been widely identified because of the short (<150 nt) read length of conventional RNA-Seq approaches. The emergence of new long-read sequencing platforms and library preparation strategies could provide the opportunity to uncover global coordination of APA and alternative splicing [161,162].

### 7.3. Isolated 3′ UTR Fragments

The presence of 3′ UTR fragments that are separated from the protein-coding regions of the mRNA has been identified in a handful of studies over the past decade (Figure 4F). Capped analysis of gene expression (CAGE)-seq and full-length cDNA-seq identified several 3′ UTR-derived RNAs which are not directly originated from transcription by RNA Pol II promoters [163]. Additionally, in situ imaging approaches have shown a divergence in 3′ UTR to CDS expression ratios across tissues suggesting cell- and tissue-specific expression of isolated 3′ UTRs [163,164]. A potential role in regulating host gene translation has been suggested based on the abundance of 3′ UTR fragments being inversely correlated with protein levels from the same gene [164]. 

How are isolated 3′ UTR fragments generated? It has been suggested that some are cleaved post-transcriptionally in the cytoplasm [165]. A recent study suggested that post-transcriptional cleavage of *Impa1* transcript occurs at an internal poly(A) site to generate 3′ UTR fragments in the cytoplasm [166]. The generation of the 3′ UTR fragments appeared to occur in axons of rat cervical ganglion neurons, but not in cell bodies [166]. In this case, 3′ UTR cleavage possibly involves the RNA surveillance machinery, as evidenced by its reliance on UPF1 [166]. Some 3′ UTR fragments are associated with ribosomes [164,167] and some have the potential to encode peptides [167]. 

Clearly, further work is needed to identify the precise mechanism(s) responsible for the generation of isolated 3′ UTR fragments. What are the molecular features of the 5′ and 3′ ends of cleaved 3′ UTRs? New library preparation strategies are needed to specifically sequence these isolated fragments. The functions of isolated fragments in the nervous system remain completely unknown, but with insight into their biogenesis and sequence features, genetic approaches to investigate their functions will surely emerge.

## 8. 3′ UTR-Associated Neurological Disorders and Behavior

Polymorphisms in 3′ UTRs are associated with neurological disorders and behavior. For example, the variable number of tandem repeat (VNTR) polymorphism in the 3′ UTR of the dopamine transporter (*DAT1*) has been associated with eating and substance use disorders, and symptom severity in Tourette syndrome [168,169]. A single nucleotide polymorphism (SNP; rs2304297) in the 3′ UTR of *CHRNA6* gene, which encodes for a nicotinic acetylcholine receptor (nAChR) subunit, has been associated with adolescent cigarette smoking, smoking cessation, and striatum volume [170,171]. A SNP in the 3′ UTR of the *SNCA* gene, which encodes α-synuclein, has also been associated with Parkinson’s disease. Heightened levels of α-synuclein is pathogenic, thus whether the polymorphisms in the 3′ UTR of *SNCA* is implicated in regulation of *SNCA* expression is of great interest [172,173,174,175]. Moreover, there might be a role for 3′ UTRs variants in AD. In this regard, 3′ UTR polymorphisms in the *APP* gene have been shown to target miR-147 and miR-20a [21]. Although the exact molecular mechanism by which this SNP has an effect on these behaviors is largely unknown, it is possible that these polymorphisms impact the expression of the host gene by disrupting regulatory elements. 

Impaired RNA granule assembly is associated with long repeat expansion disorders such as myotonic dystrophy, amyotrophic lateral sclerosis, and spinocerebellar ataxia [176,177]. Repeat expansion in 3′ UTRs can sequester RBPs into granules affecting their normal functions. For instance, in the debilitating neuromuscular disorder Myotonic dystrophy type I, a repeat expansion in the 3′ UTR of the *DMPK* gene sequesters the splicing factor MBNL1 into nuclear RNA foci to prevent from functioning in normal pre-mRNA processing [178,179].

In Huntington’s disease (HD), expression of many alternative 3′ UTR isoforms, among others the huntingtin (*HTT*) gene, are altered. Profiling of alternative 3′ UTR isoforms has shown that *HTT* long 3′ UTR isoform specifically is altered in multiple tissues of HD patients [180]. The exact mechanism whereby the altered abundance of the long 3′ UTR isoform of *HTT* contributes to HD pathology remains elusive. 

Strong evidence for the contribution of 3′ UTR mutations in neurological disease susceptibility and pathogenesis is still limited. Increased whole genome sequencing depth and sensitivity, increased numbers of sequenced individuals, and improved 3′ UTR annotations might lead to new discoveries of medically relevant 3′ UTR SNPs. The use of massively parallel reporter assays to assess the role of 3′ UTR SNPs on translational control could be a useful approach to uncover whether particular 3′ UTR SNPs have functional relevance, such as has been performed to identify human genetic variants that influence splicing [181].

## 9. Conclusions

The 3′ UTR imparts post-transcriptional regulation by impacting mRNA stability, subcellular localization, and translational control. APA controls the susceptibility of transcripts to this regulation, and the functional roles for alternative 3′ UTR isoforms have been discovered for only a handful of genes. New emerging mechanisms suggest that 3′ UTRs have roles in modulating the protein interactome of newly translated proteins and coordinating alternative splicing. Given the limitations of some neuronal-like immortalized cultured systems, we call for an increased emphasis on performing 3′ UTR functional studies in primary neuronal culture and whole organisms. 

## Abbreviations

AD	Alzheimer’s disease
AP	Action potential
APA	Alternative polyadenylation
ARE	AU-rich element
BONCAT	Bio-orthogonal non-canonical amino acid tagging
CA1	Cornu ammonis 1
CDS	Coding sequence
CRISPR	Clustered regularly interspaced short palindromic repeats
DRG	Dorsal root ganglion
EPSP	Excitatory postsynaptic potential
FISH	Fluorescence in situ hybridization
FP	Fluorescent protein
FUNCAT	Fluorescent noncanonical amino acid tagging
HD	Huntington’s disease
L-VGCC	L-type voltage-gated calcium channel
m^6^A	N6-methyladenosine
miRNA	microRNA
mRNP	messenger ribonucleoproteins
MS2	MS2 bacteriophage coat protein
pA	Poly(A) site
PAS	Poly(A) signal
poly(A)	Polyadenylation
Puro	Puromycin
RBP	RNA-binding protein
RNA-Seq	RNA-sequencing
shRNA	Short hairpin RNA
SNP	Single nucleotide polymorphism
UTR	Untranslated region
VNTR	Variable number of tandem repeats
λN	N protein of bacteriophage λ
